# Spatially and temporally resolved gas distributions around heterogeneous catalysts using infrared planar laser-induced fluorescence

**DOI:** 10.1038/ncomms8076

**Published:** 2015-05-08

**Authors:** Johan Zetterberg, Sara Blomberg, Johan Gustafson, Jonas Evertsson, Jianfeng Zhou, Emma C. Adams, Per-Anders Carlsson, Marcus Aldén, Edvin Lundgren

**Affiliations:** 1 Division of Combustion Physics, Lund University, Lund 221 00, Sweden; 2 Division of Synchrotron Radiation Research, Lund University, Lund 221 00, Sweden; 3 Competence Centre for Catalysis, Chalmers University of Technology, 412 96 Göteborg, Sweden

## Abstract

Visualizing and measuring the gas distribution in close proximity to a working catalyst is crucial for understanding how the catalytic activity depends on the structure of the catalyst. However, existing methods are not able to fully determine the gas distribution during a catalytic process. Here we report on how the distribution of a gas during a catalytic reaction can be imaged *in situ* with high spatial (400 μm) and temporal (15 μs) resolution using infrared planar laser-induced fluorescence. The technique is demonstrated by monitoring, in real-time, the distribution of carbon dioxide during catalytic oxidation of carbon monoxide above powder catalysts. Furthermore, we demonstrate the versatility and potential of the technique in catalysis research by providing a proof-of-principle demonstration of how the activity of several catalysts can be measured simultaneously, either in the same reactor chamber, or in parallel, in different reactor tubes.

## Abstract (web summary)

Visualization of the gas distribution around working catalyst is crucial for understanding structure–activity relationships. Here, the authors show that gas distribution can be imaged *in situ* with high spatial and temporal resolution using infrared planar laser-induced fluorescence.

## Graphical abstract

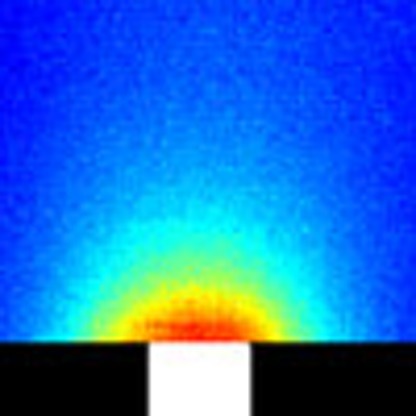

Catalysis research is highly relevant to society because industrial production of chemicals and environmental protection by pollution control rely heavily on (heterogeneous) catalyst technologies^1^. In this context the oxidation of carbon monoxide (CO) on Pt-group metals is one of the most-studied prototypical catalytic reactions because of its practical relevance and general applicability. Despite its apparent simplicity, the reaction is challenging to study and describe because of peculiar kinetics such as bistability and oscillations, and fascinating spatiotemporal behaviour^2^.

In the case of industrial catalyst development, the efficiency of a material to catalyse a particular reaction is evaluated by analysing the end products, after the reactants have passed the catalyst containing the active material, using appropriate characterization techniques, such as mass spectrometry (MS), gas chromatography (GC), Fourier transform infrared spectrometry (FTIR) or specific analysers depending on the reaction. However, for model catalysts, such as single crystals, used to yield an understanding of the surface chemistry and structure, the evaluation is carried out by, for example, investigating catalytically active sites on the atomic scale, usually at ultrahigh vacuum. At more realistic and industrial-like operating conditions in the mbar pressure region and above, the gas-phase situation becomes more complicated. This is because in such investigations the reactants pass over the catalyst, causing gas-phase phenomena such as mass-transfer limitation and convection, which changes the conditions near the catalyst. In fact, this may induce the formation of surface structures not related to the catalytic processes in conjunction with a change in the catalytic activity, yielding an intense debate on the active sites or even the active phase^3^,^4^.

Spatially resolved measurements with high quality using traditional analytics (MS, GC and FTIR) have been pursued in earlier studies. For instance, in the work described in refs 5, 6, 7 a scanning mass spectrometer with a capillary probe that can stepwise (2 s per step in ref. 5) be scanned over the sample was used. This approach allows for pointwise spatially resolved measurements but is unable to deliver two-dimensional (2D) measurements on a subsecond scale to catch dynamic changes in the gas phase. Furthermore, probing techniques are inherently intrusive and might affect the gas flow and temperature above the catalyst disturbing the dynamics. Another example of a technique that is traditionally used is FTIR^8^,^9^,^10^,^11^, a nonintrusive method that has been extended to two dimensions for combinatorial screening of catalysts with a stated detection limit of 1,000 p.p.m. for propane^11^ and 1% of CO_2_ with a collection time of 17 s (ref. 12). However, FTIR is a so-called line-of-sight technique that relies on the absorption along a path, making it unable to resolve the gas phase in the third dimension and when temperatures are inhomogeneous in the integration path, the spectral signal is distorted. The integration time also limits the temporal resolution to ∼2 s per scan^13^. Nonintrusive laser-based techniques, such as Raman scattering, have also been successfully applied to catalysis studies^14^,^15^,^16^,^17^. The difficulty with Raman scattering is the low cross-section, limiting the measurements to one-dimension (1D) and most often with long collection times because of averaging, ranging from several minutes to an hour (although in other fields, and with a very sophisticated experimental set-up, single shot Raman has been achieved^18^). This makes it mostly suitable for stationary situations where changes in the gas-phase conditions are minimal or very slow. The mentioned techniques have the capability for multispecies detection, but are all limited either in the temporal or the spatial domain and are therefore less suitable for measurements of the 2D gas distributions above catalytically active surfaces in a changing environment.

Laser-induced fluorescence (LIF) has been used in numerous research fields, such as combustion and medical research^19^,^20^, to measure temperature, velocity and the concentration of a probed gas. LIF has high spatial and temporal resolution, is nonintrusive and can be used to perform 2D measurements *in situ*, without disturbing the flow properties of the gas^21^. It is then often referred to as planar laser-induced fluorescence (PLIF).

Although recognized early as a potential method for catalysis research^22^,^23^,^24^,^25^, its use has been very limited and restricted to the ulraviolet/visible spectral regime, meaning several gases important for catalysis, such as CO_2_, CO and small hydrocarbons^26^,^27^, have remained elusive because of excitation and detection limitations. However, the technical development during recent years in lasers and infrared detection capabilities^28^ together with efficient detection and background suppression schemes developed in an earlier study^29^ enables the use of PLIF for several of these previously undetectable gases. The first detection of CO_2_ to study catalysis was made in a recent study by Zetterberg *et al*.^29^; however, it was limited to averaged data, lacking the capability of capturing single shot data with sufficient signal-to-noise to really make an impact. However, with further refinements of the experimental set-up and methodology we have been able to use infrared PLIF to capture instantaneous, 2D images of the probed CO_2_ gas formed during oxidation of CO over porous supported noble metal catalysts. A schematic of the process is shown in Fig. 1. In this report we highlight the advantage of being able to perform spatially resolved, *in situ* measurements of the gas phase close to catalysts by means of infrared PLIF. First, we show how the catalytic ignition and extinction of the CO oxidation can be followed for a powder catalyst with a frame rate of 10 Hz, directly visualizing the change in the gas composition close to the catalyst surface. Second, the possibility to measure the CO_2_ distribution above two catalysts that are simultaneously situated in the chamber is presented, and how the presence of the two samples affects each other is discussed. Finally, we show that by physically separating the catalytic samples, the differences in activation temperatures can be directly visualized, and activation energies extracted. The study sheds light on several aspects for catalysis research, especially the possibility to visualize an event, in this case the elusive CO_2_ product, with high temporal and spatial resolution, at realistic industrial conditions.

## Results

### Experimental description

In our experiments we used infrared PLIF to image the temperature-dependent dynamics of CO_2_ close to the surface of catalytic discs during ignition and extinction of catalytic CO oxidation. The samples studied were industrial-like pressed noble metal powder catalysts, and we specifically investigated how the gas distribution around the catalytic discs was changing with sample position, as well as in the presence of two samples in the chamber. In the end we give an example of how to perform combinatorial measurements on three samples simultaneously, without having the samples affecting each other, as a way of screening potential catalysts in the development phase both academically and industrially.

In all our measurements a mass spectrometer, connected to the outlet of the reactor via a leak valve, was used to monitor the global gas composition. The MS signals have a time lag of ∼5 s in the present set-up, compared with the LIF signal (which is almost instantaneous), and the increase in the CO_2_ signal as the sample ignites is slower. The lag originates from the time it takes for the gas to reach the MS and is dependent on the flow speed, pressure and the volume of the gas has to pass to reach the leak valve.

### Measuring CO_2_ distributions over single-catalyst samples

We first investigated the CO_2_ distribution over two samples, 2%Pd/CeO_2_ (hereafter called the Pd-sample) and 2%Pt1%Pd/CeO_2_ (hereafter called the Pt-Pd-sample). Worth noting is that the percentages given is by weight, meaning that the concentration of active atoms is the same in both samples. To that end we took infrared PLIF snapshots of the CO_2_ distribution around each catalyst (Fig. 2a,b and Supplementary Movies 1 and 2). The gas flows were 18  ml_*n*_ min^−1^ CO, 18 ml_*n*_ min^−1^ O_2_ and 36 ml_*n*_ min^−1^ Ar at a total pressure of 105 mbar. The temperatures discussed in the text refer to the thermocouple temperatures.

Already at 137 °C (time I, Fig. 2a,d) the fluorescence signal close to the sample surface increased slightly, indicating the presence of CO_2_, as both samples started to ignite. At 165 °C the Pd-sample ignited (time II, Fig. 2b), which was manifested as a circularly shaped CO_2_ distribution above the sample that decreased away from the sample surface. This is expected because the CO_2_ distribution around the sample is mainly determined by diffusion. In contrast, the fluorescence measured above the Pt-Pd-sample, while stronger than that measured at 137 °C, was still low at this temperature, thus providing evidence for a low catalytic activity (time II, Fig. 2e). Only when the temperature was ramped up to 220 °C the Pt-Pd-sample ignited, resulting in a circularly shaped CO_2_ distribution similar to the one observed for the Pd-sample at 165 °C (time III, Fig. 2f).

Above these temperatures, both samples reached a steady-state CO_2_ production, which resulted in only small changes in the distribution of the gas. A slight drag towards the right can be seen, which can be attributed to the gas flow passing from left to right in the image. At 250 °C, the samples reached equilibrium, the gas distribution was steady and the CO_2_ concentration had stopped to increase.

The fluorescence signal was extracted 1 mm above each sample's surface from each snapshot image (Fig. 2g). Furthermore, we measured the MS signal—normalized to the known initial partial pressure of CO and O_2_; CO_2_ was scaled to match the observed conversion of CO—to elucidate the signal's dependence on temperature (Fig. 2h,i), and the temperature of the samples' surface and sample holder (Fig. 2j).

One way to investigate the catalytic activity is to study the spatial gradient vertically away from the Pd-sample, something infrared PLIF gives the opportunity to do on a single-shot basis. The gradient was followed in time as a function of temperature (Fig. 2k). From the image it can then be seen that already in the kinetically controlled regime, at 100 °C, there is a significantly higher CO_2_ concentration close to the surface than further away. Just before ignition the partial pressure of CO_2_ ranges from ∼1.5 mbar at 0.2 mm above the surface to 0.8 mbar at 2 mm from the surface (the latter being close to the pressure observed by the MS at the outlet of the reactor).

For both samples, the concentration plateaus both in the fluorescence and in the MS signal show that the reactions are limited by the CO diffusion to the catalyst, which occurs almost immediately after ignition, and that all the CO molecules that reach the surface are oxidized^30^.

When the temperature was ramped down the signals concomitantly decreased as the catalytic activity of the samples decreased. The temperature at which the Pd-sample extinguishes is lower (133 °C) than the corresponding temperature at which the Pt–Pd extinguishes (199 °C), which mimics the corresponding trend in the ignition process where corresponding temperatures are 165 and 218 °C, respectively. The difference in temperatures for ignition and extinction is in agreement with previous studies of supported Pd and Pt catalysts^31^.

In general, the averaged infrared PLIF signals (Fig. 2g) were in agreement with the ones obtained by MS (Fig. 2h,i), with only a slight delay of the latter relative to the former because of the time required for the gas distribution around the sample to reach the MS. The instant at which the samples became active also coincided with an increase in the surface temperature of the samples because of the exothermic nature of the reaction (Fig. 2j). Although the accuracy of the measured absolute temperatures was ∼10–20 °C, these measurements provided an additional validation and understanding of the dynamics of the gas distribution during the experiment. The temperature measurements also did not suffer from the time-lag associated with the MS measurement, thus making a more accurate measurement of the activation temperatures possible. In the present case, the ignition can in principle be monitored by the thermocouple or better the infrared camera; however, for many other reactions (or for low concentrations of CO), the reaction is not as strongly exothermic as in the case of CO oxidation, disqualifying the thermocouple reading as a probe for the ignition.

### Simultaneous measurements of CO_2_ above two samples

To demonstrate the versatility and high-resolution measurement capabilities of infrared PLIF, we next measured the CO_2_ distribution over the two samples discussed in the previous section, simultaneously and under the same conditions as above (18 ml_*n*_ min^−i^ CO, 18 ml_*n*_ min^−1^ O_2_ and 36 ml_*n*_ min^−1^ Ar at a total pressure of 105 mbar). To that end, we placed the two catalytic samples next to each other in the reactor (∼8 mm apart) and measured and analysed the infrared PLIF signal, showing that it is possible to distinguish the CO_2_ distribution around each sample.

The results of the temperature-dependent infrared PLIF measurements are shown in Fig. 3, alongside measurements of the MS signal, and the temperatures of the samples' surfaces and holder. We found minimal activity already at *T*=136 °C (Fig. 3a) and the Pd-sample ignited at a temperature of 170 °C (Fig. 3b), resulting in the formation of a cloud-like CO_2_ distribution similar to the one observed in the single-sample measurements. The spatial resolution of the experimental set-up is high enough to spatially distinguish which of the two samples that becomes active by probing the corresponding CO_2_ distribution. As the temperature was further increased, the Pt–Pd sample also became active at ∼221 °C (Fig. 3c), and a much larger cloud of CO_2_ is now visible above the surface of the sample holder. However, the distributions are still highly localized and separable above each of the samples

At 250 °C (Fig. 3d), both samples reached equilibrium and the gas distribution around the samples was found to change very little with further increase in the temperature. The CO_2_ concentration in the chamber was inhomogeneous, with a somewhat stronger fluorescence signal detectable on the right-hand side of the reactor, which can be attributed to the gas flowing through the reactor from left-to-right.

When the temperature was ramped down, the Pt–Pd sample was first to extinguish (Fig. 3e) soon thereafter followed by extinction of the Pd-sample (Fig. 3f). The ignition/extinction events for both samples give rise to corresponding signatures in the LIF trace, MS signal and temperature data (Fig. 3g–i). This experiment shows the potential of laser diagnostics and the advantage of having 2D, spatially resolved, nonintrusive measurements of the gas distribution, opening up for simultaneous characterization of more than one sample at a time and direct *in situ* comparisons, something very useful, for example, when studying systems where more than one active catalyst is needed to drive different reactions, and how the presence of one affects the other.

### Probing the CO_2_ production from three samples in parallel

Because of the possible interaction of the samples as demonstrated above, we also applied and demonstrated the versatility of the technique by probing the CO_2_ production originating from three separate samples simultaneously and, in parallel, by placing these into three separated flow tubes. We pursued this solution for situations in which having more than one sample in the reactor changes the reaction of the adjacent samples and *vice versa*. To address this problem we developed our reactor to have the possibility to include three tubes (see Methods section). The experimental set-up used the same gas system as in the experiments on one and two samples, the only modification being that the gas was directed to flow through the three tubes. The tubes ended inside the middle of the vacuum chamber where the laser sheet was placed as close as possible (<1 mm) to the exits of the tubes. In this way the gas originating from each individual tube could be probed simultaneously by the laser. In each of the three tubes a monolith sample was placed, each containing Pd/CeO_2_, Pt/CeO_2_ and Pt/Al_2_O_3_, respectively.

We performed similar measurements, as with the one- and two-sample cases, of the infrared PLIF, MS and temperature of the samples, as a function of temperature (Fig. 4), but at higher pressure, 1 bar.

In contrast to our previous collection of snapshot images of the infrared PLIF, we here show averaged (over 10 laser pulses) infrared PLIF images that are measured at the exit of the three tubes (Fig. 4a–d). At 70 °C (Fig. 4a) none of the samples had ignited and no infrared PLIF signal was visible. As the temperature was increased, the Pd/CeO_2_ sample ignited at 180 °C (Fig. 4b), followed by the Pt/CeO_2_ at 278 °C (Fig. 4c) and Pt/ Al_2_O_3_ at 352 °C (Fig. 4d). The signals were not centred in the tubes because of the low flow out from the tubes and because the outlet was placed at the bottom of the reactor.

The onset of ignition for each of the samples yielded an abrupt increase in the averaged infrared PLIF signals at the exit of the tubes (Fig. 4e). This contrasts the MS results where it is not possible to extract the ignition times and temperatures for the different samples (Fig. 4f). While the MS signal changes with temperature, the changes are not as pronounced as in the infrared PLIF data, which makes it impossible to tell from which sample the signal originates. This also opens up for the possibility of extracting quantitative data for the individual catalysts. Activation energies are calculated from the three Arrhenius plots (Fig. 4h) that are extracted from the infrared PLIF signals (Fig. 4g). As expected, the activation energy of 0.25 and 0.53 eV found for the two CeO_2_-supported samples are lower than for the Pt supported by Al_2_O_3_ (1.05 eV). This is also in good agreement with what is reported in literature^32^,^33^. In contrast, the overlapping CO_2_ MS signals complicate the analysis of finding the individual reaction rates for each of the three catalysts and thus constrains the possibility to achieve the activation energy with the MS. The infrared PLIF method could therefore be used to study several catalysts in parallel in a way that has not been possible before.

## Discussion

The present report shows that the technical development of lasers, detectors and data treatment during the last 15 years enables direct 2D detection of small molecules relevant for catalysis previously not attainable by LIF. To detect CO_2_ at elevated temperatures and realistic gas conditions, it is necessary to excite the molecule with a wavelength of 2.7 μm, demanding a high-energy laser with a respectable power and narrow linewidth. Further, a fast gateable infrared camera with the ability to detect the fluorescence light at 4.3 μm is needed, since a fast subtraction scheme is required to remove the otherwise completely dominating thermal background, a short gate time (ns–μs regime) and a high enough frame rate makes this possible. Thus, both laser source and detector used in the present investigations are nonstandard making the combination truly unusual, and have not previously been used for catalysis-related studies.

With PLIF, whether in the infrared or ulraviolet/visible regime, the gas distribution around a catalyst can be visualized with high spatial and temporal resolution (below 500 μm and down to ∼50 μm depending on excitation wavelength and from 10 μs down to below 10 ns for a single snapshot) with the only disturbance being the excitation of the interrogated species. For the direct product of CO oxidation, this has not been possible until now. The results presented here is an important step for a better understanding of gas phase-related phenomena in catalysis, where dynamics can be studied that are otherwise often hidden in averaged data.

The study implies several new aspects for catalysis research, for example, with PLIF it is possible to measure the gas surrounding the sample at realistic pressures, revealing the actual gas composition close to the surface previously not attainable, for a correct interpretation of the active site or phase. The study also shows with images that by following the gas distribution in time it is possible to distinguish whether the entire catalyst or only part of it is active, or by directly comparing two catalysts decide which has the lowest activation temperature.

By combining the present gas phase measurements with simultaneous X-ray diffraction measurements^34^, the correct active site/phase should become available. In this way, it should be possible to determine the surface structure and the gas composition close to the surface of the catalyst, previously simultaneously unattainable properties. Furthermore, our study shows how PLIF applied to catalysis has a potential for simultaneous characterization or combinatorial studies of industrial catalysts in a straightforward way. While infrared PLIF applied to catalysis is just barely developed, a present drawback of optical and laser-based methods is the need for specialized reactors with optical access with at least one, but often as many as three transparent windows. Further, the present relatively complicated experimental set-up and data analysis limits the use to experienced and trained researchers.

To this end, we are in the process of developing more standardized reactors, lasers and software suitable for (infrared) PLIF, with the possibility of applying an additional experimental technique. An example of a step in this direction is the development of a reactor towards simultaneous synchrotron radiation and laser measurements, where surface and gas phase information can be extracted simultaneously under the same conditions and at the same sample.

Using modern laser and detection techniques (not limited to the infrared regime), the experiments presented here can be extended to a vast number of gas species, such as CO, CH_4_, NH_3_ and NO. Our present experiment highlights the principle of the experiment and the spatial resolution provided applying infrared PLIF to catalysis, where flows and gas distributions can be measured instantaneously in 2D, something conventional techniques such as MS, GC and FTIR lacks, and provides a tool that in principle can be used simultaneously with synchrotron-based measurements.

## Methods

### Infrared PLIF

**Figure 1:**
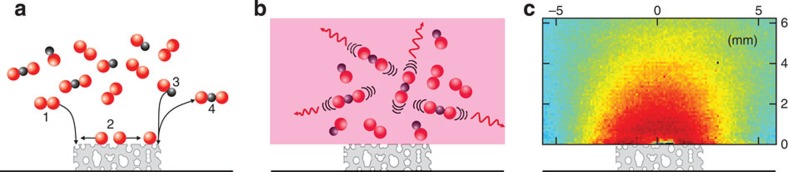
Schematic of planar laser-induced fluorescence. Schematic of PLIF measurements of the activity of a model catalyst (**a**) shows the adsorption and dissociation of O_2_ on the catalyst at points **1** and **2**, respectively, and the adsorption of CO and production of CO_2_ at points **3** and **4**, respectively. (**b**) The laser sheet (pink) that excites the CO_2_ molecules above the catalyst; the fluorescence from the excited molecules is detected. An example of the CO_2_ distribution above an active catalyst at elevated temperature and realistic pressure is shown in **c**.

**Figure 2:**
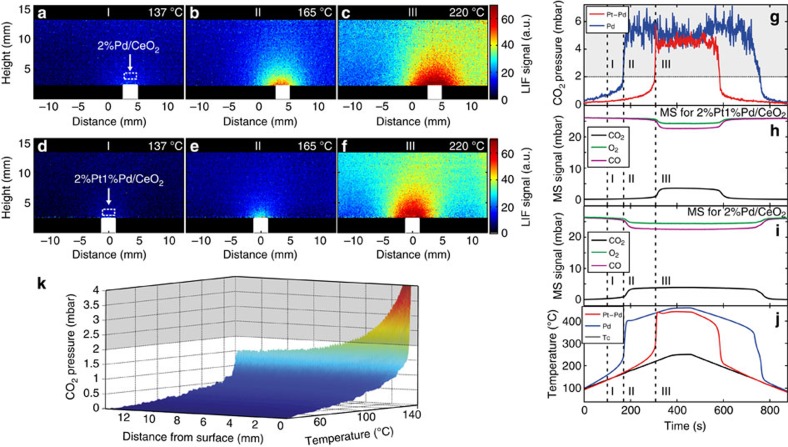
CO_2_ distribution over two single-catalyst samples. CO_2_ distribution over two single-catalyst samples at different temperatures at a total pressure of 105 mbar, infrared PLIF single-shot images of the CO_2_ distribution over (**a**–**c**) Pd sample and (**d**–**f**) Pt-Pd-sample surface at different temperatures (see also Supplementary Movies 1 and 2). For both samples, we also show (**g**) the fluorescence signals measured 1 mm over the surface of the samples, calibrated to CO_2_ partial pressure below the shaded area, (**h**,**i**) the calibrated MS profile of CO, O_2_ and CO_2_ for the Pt–Pd and Pd samples, respectively, (**j**) the temperature of the samples' surface (red and blue) and sample holder (*T*c) and (**k**) the CO_2_ gradient (calibrated below the shaded area) from ∼0.2 mm above the sample as a function of temperature. The roman numerals indicate times during the experimental measurement that correspond to the time when the snapshots shown in Fig. 2a–f were recorded.

**Figure 3:**
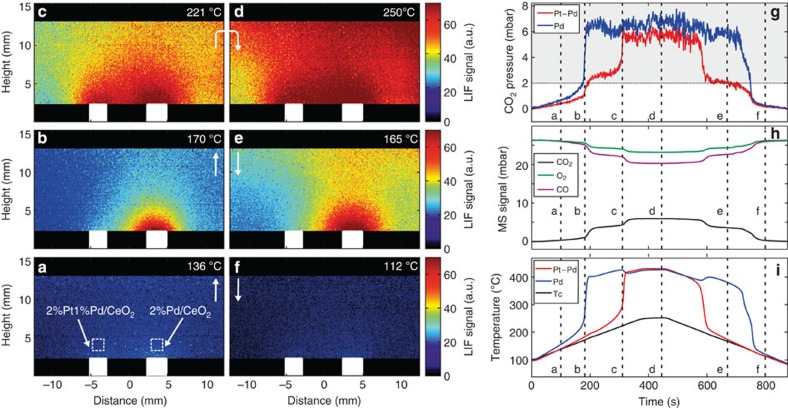
The CO_2_ distribution over two samples simultaneously positioned in the reactor. (**a**–**f**) Infrared PLIF single-shot images during the reaction showing the CO_2_ distribution over a Pt–Pd sample and a Pd powder catalyst surfaces at different times and temperatures and at 105 mbar of total pressure (see Supplementary Movies 3). (**g**) The fluorescence signal 0.7 mm above each sample (red and blue). (**h**) The MS signal. (**i**) The temperature of the sample surfaces (red and blue) measured with the infrared camera together with the temperature of the sample holder, measured by a thermocouple (*T*c).

**Figure 4:**
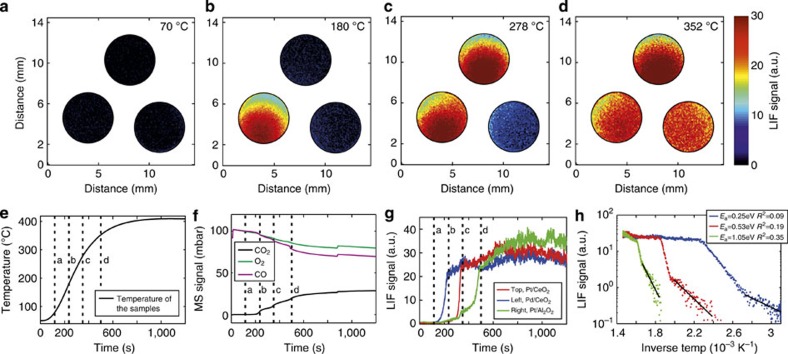
The CO_2_ signal from three different samples in three separate exhaust tubes. (**a**–**d**) The CO_2_ signal from the gas exiting the three tube ends are shown in the top panel (see Supplementary Movie 4). (**e**) The temperature measured by the thermocouple located centred between the tubes at the same position as the samples, (**f**) the MS signal during the event and (**g**) the averaged infrared PLIF signal exiting each tube, which is also shown in three Arrhenius plots in **h**.

The principles of infrared PLIF are (Fig. 1) as follows: (1) a laser beam excites a vibrational/rotational level in the molecule of interest; (2) the molecule fluoresces in response to the laser excitation by emitting light at a wavelength that is known *a priori*; (3) the emitted fluorescence is detected and analysed. In our study, the infrared laser beam was generated by difference-frequency mixing the output from a dye laser (Sirah PRSC-D-18) at 763 nm with the fundamental frequency from a Nd:YAG laser (Spectra Physics, PRO 290-10) at 1,064 nm in a LiNbO_3_ crystal, yielding a 2.7-μm laser pulse (with a pulse energy of 4 mJ, 5 ns pulse duration and the linewidth estimated to 0.025 cm^−1^)^35^. The output from the laser was used to probe the P(12) line of the (00^0^0)→(10^0^01) combination band of the CO_2_ molecules in the vicinity of the catalyst, and the fluorescence at 4.3 μm was detected by a 2D infrared camera (Santa Barbara Focal Plane, SBF LP134). The infrared PLIF images visualize the CO_2_ distribution (Fig. 1c) in a 25 × 13-mm^2^-sized rectangle above the catalyst. The images were collected every 0.1 s and with a temporal resolution for each frame, limited by the exposure time of the camera, of 15 μs, and the spatial resolution was ∼400 μm and limited by the thickness of the lasersheet. The detection limit for CO_2_ was estimated to 100 p.p.m. (or 0.1 mbar) in the present measurements. A schematic describing the laser set-up, optics and the collection of the images is shown in the Supplementary Fig. 1. The infrared background was subtracted with a scheme presented in ref. 29.

The CO_2_ fluorescence intensity has been calibrated to mbar for concentrations lower than ∼2 mbar. This is made possible by making calibration measurements at known CO_2_ concentrations and temperatures, creating a calibration curve. As the temperature field in the reactor is inhomogeneous, it has to be corrected for in order to give a correct representation of the CO_2_ concentration. Infrared PLIF images with a homogeneous CO_2_ distribution is therefore collected at temperatures matching those of the experimental data. While the fluorescence signal is temperature-dependent, these images represent the temperature field and can be used to compensate the measurement data. In this way not only the density differences due to temperature is taken into account but also the difference in population. The dependence of fluorescence signal on temperature is covered more thoroughly in the Supplementary Methods section and is demonstrated in Supplementary Fig. 2.

### Reaction chamber

The experiments were carried out in a cubical stainless steel reaction cell with a total volume of 240 ml. The samples were placed on a Mo sample holder in the middle of the reactor. The samples could be viewed in the reactor from four different directions through four CaF_2_ windows. The Mo sample plate was heated by a boralectric heater (Métaux Céramiques Systèmes Engineering; BN) held by two Ta rods that were attached to current feed-throughs and connected to a 20-A power supply. The samples were thus indirectly heated via the Mo plate and the temperature of the sample holder was measured with type-C thermocouples attached to the Mo plate. The power supply was regulated via a LabView programme in order to increase and decrease the temperature in a controlled way. A schematic of the experimental set-up is shown in the Supplementary Fig 1.

The surface temperature of the samples was measured using an infrared camera (FLIRP620); however, as the emissivity changes with temperature, it is hard to get an absolute temperature from this measurement. It is, however, a very good indicator of when the sample ignites.

The gas flows were 18 ml_*n*_ min^−1^ CO, 18 ml_*n*_ min^−1^ O_2_ and 36 ml_*n*_ min^−1^ Ar at a pressure of 105 mbar for the first two experiments with one and two samples in the reactor.

A premixed 10% CO_2_ in Ar was used for calibration purposes. The gases were introduced into the reactor cell via individual Bronkhorst mass-flow controllers (Bronkhorst EL-FLOW, 50 mln min ^−1^) that can vary the gas flow from 1 to 50 ml min^−1^. To keep the pressure constant, a pressure controller (Bronkhorst EL-PRESS) was attached to the gas outlet. The gas composition was studied by a quadruple mass spectrometer (Pfeiffer PrismaPlus QMG220), connected to the reactor outlet via a leak valve.

### The samples

The supported noble-metal catalysts were prepared by incipient wetness impregnation targeting the same molar amount of noble metal atoms. The alumina (Puralox SBa 200, Sasol) and ceria (99.5 H.S.A. 514, Rhône-Poulenc) support materials were first thermally stabilized by a treatment in air at 600 °C for 2 h. Each support was then dispersed in an aqueous solution consisting of distilled water and the relevant concentration of noble metal precursor, tetraammineplatinum(II)nitrate (4.0 wt.% (NH_3_)_4_Pt(NO_3_)_2_, Alfa Aesar GmbH & Co. KG) and/or tetraamminepalladium(II)nitrate (4.6 wt.% (NH_3_)_4_ Pd(NO_3_)_2_, Alfa Aesar GmbH & Co. KG) to obtain the desired noble metal loading, that is, 4%Pt/alumina, 4%Pt/ceria, 2.2%Pd/ceria and 1.1 Pd%–2%Pt/ceria (% refers here weight percentage). For the co-impregnation of platinum and palladium, the noble metal precursors were completely mixed before impregnation. To increase the interaction between the noble metal complex and the support, the pH of the solution was adjusted by NH_4_OH addition taking into account the isoelectric point of each oxide. The obtained slurry was then stirred for 20 min, frozen with liquid nitrogen and freeze-dried for 12 h to preserve high noble metal dispersion. The resulting powder was finally calcined in air at 550 °C for 1 h using a heating rate of 5 °C min^−1^ starting from room temperature. The total surface area of the powder samples was measured with N_2_ physisorption at 77 K (Micromeritics Tristar). Using the BET method for *P*/*P*_0_=0.05–0.20, the specific surface area was calculated to be 173, 156, 159 and 161 m^2^ g^−1^ for the 4%Pt/alumina, 4%Pt/ceria, 2.2%Pd/ceria and 1.1 Pd%–2%Pt/ceria, respectively.

### The tube reactor set-up

A schematic of the experimental set-up is shown in Supplementary Fig. 3. The tube reactor consists of an outer fused silica (FS) tube with three smaller FS tubes inside. The larger tube has a diameter of 16 mm and is fitted with a CF16 end to connect to the cubical reaction cell described above, and the smaller tubes are 500 mm long and have an inner diameter of 5 mm. The smaller tubes are placed in such a way that they end in the middle of the cubical reaction cell. The reactor was heated by a resistance heating-wire coiled around the larger tube and the temperature measured with a type-K thermocouple placed in the centre of the larger tube between the smaller tubes. The CF16 end of the larger tube was used to connect to the CF40 cube and the smaller tubes were placed such that they ended in the middle of the cube.

The gas was supplied through the large tube, in which the gas was divided up into the smaller tubes where monolith samples (20 mm long and 3-mm thick 2 × 2 channels monolith impregnated with noble metal) were placed. The space between the large and the small tubes were sealed with quartz fibres, to prevent the gas from flowing outside the small tubes. The small tubes ended centred in the reaction cell and the laser sheet was placed as close as possible to the tube ends, in order to probe the exiting gas. The gases were regulated and pressure-controlled in the same way as in the measurements with one and two samples. The flow fed through the tubes consisted of 5 ml_*n*_ min^−1^ CO, 5 ml_*n*_ min^−1^ O_2_ and 40 ml_*n*_ min^−1^ Ar at a pressure of 1,000 mbar.

## Author contributions

J.Z.E. performed the measurements and did the data evaluation together with S.B. and had the major responsibility for preparing the paper (including SI), J.E. designed the reactor for combinatorial studies and took part in and evaluated those measurements. J.G. designed the reactor and took part in the development of the analysis methods, E.L. and P.A.C. wrote part of the paper. J.Z.H. made calibration measurements for quantitative CO_2_ profiles. E.C.A. prepared the samples, and M.A. and E.L. supervised the project.

## Additional Information

**How to cite this article**: Zetterberg, J. *et al*. Spatially and temporally resolved gas distributions around heterogeneous catalysts using infrared planar laser-induced fluorescence. *Nat. Commun*. 6:7076 doi: 10.1038/ncomms8076 (2015).

## Supplementary Material

Supplementary Figures, Supplementary Methods and Supplementary References. Supplementary Figures 1-3, Supplementary Methods and Supplementary References.

Supplementary Movie 1 Movie of the CO_2_ IR PLIF signal for a single sample 2%Pd/CeO_2_, the LIF signal is partly calibrated and is an average of 10 laser shots.

Supplementary Movie 2 Movie of the CO_2_ IR PLIF signal for a single 2%Pt1%Pd/CeO_2_ sample. The LIF signal is partly calibrated and is an average of 10 laser shots.

Supplementary Movie 3 Movie of the CO_2_ IR PLIF signal for both samples simultaneously, the LIF signal is partly calibrated and is an average of 10 laser shots.

Supplementary Movie 4 Movie of the CO_2_ IR PLIF signal at the exit of the different tubes in the tube reactor experiments, the LIF signal is not calibrated and is an average of 10 laser shots.

## References

[b1] Heck R. M., Farrauto R. J. & Gulati S. T. Catalytic Air Pollution Control: Commercial Technology. 3rd edn John Wiley (2009).

[b2] Ertl G. Handbook Of Heterogeneous Catalysis 2nd edn (Wiley-VCH (2008).

[b3] van Rijn R. . Comment on "CO oxidation on Pt-group metals from ultrahigh vacuum to near atmospheric pressures. 2. palladium and platinum”. J. Phys. Chem. C 114, 6875–6876 (2010).

[b4] Gao F., Wang Y. L. & Goodman D. W. Reply to "Comment on 'CO Oxidation on Pt-Group Metals from Ultrahigh Vacuum to Near Atmospheric Pressures. 2. Palladium and Platinum”. J. Phys. Chem. C 114, 6874–6874 (2010).

[b5] Roos M. . Scanning mass spectrometer for quantitative reaction studies on catalytically active microstructures. Rev. Sci. Instrum. 78, 084104 (2007). 17764340 10.1063/1.2777167

[b6] Sidwell R. W., Zhu H. Y., Kee R. J. & Wickham D. T. Catalytic combustion of premixed methane-in-air on a high-temperature hexaaluminate stagnation surface. Combust. Flame 134, 55–66 (2003).

[b7] Sidwell R. W. . Catalytic combustion of premixed methane/air on a palladium-substituted hexaluminate stagnation surface. Proc. Combust. Inst. 29, 1013–1020 (2002).

[b8] Snively C. M., Katzenberger S., Oskarsdottir G. & Lauterbach J. Fourier-transform infrared imaging using a rapid-scan spectrometer. Opt. Lett. 24, 1841–1843 (1999). 18079949 10.1364/ol.24.001841

[b9] Snively C. M., Oskarsdottir G. & Lauterbach J. Chemically sensitive parallel analysis of combinatorial catalyst libraries. Catal. Today 67, 357–368 (2001).

[b10] Tan C. K. C., Delgass W. N. & Baertsch C. D. Spatially resolved in situ FTIR analysis of CO adsorption and reaction on Pt/SiO_2_ in a silicon microreactor. Appl. Catal. B Environ. 93, 66–74 (2009).

[b11] Snively C. M., Oskarsdottir G. & Lauterbach J. Parallel analysis of the reaction products from combinatorial catalyst libraries. Angew. Chem. Int. Ed. 40, 3028–3030 (2001). 10.1002/1521-3773(20010817)40:16<3028::AID-ANIE3028>3.0.CO;2-X 12203638

[b12] Snively C. M. & Lauterbach J. Sampling accessories for the high-throughput analysis of combinatorial libraries using spectral imaging. Spectroscopy 17, 26–33 (2002).

[b13] Hendershot R. J., Fanson P. T., Snively C. M. & Lauterbach J. A. High-throughput catalytic science: parallel analysis of transients in catalytic reactions. Angew. Chem. Int. Ed. 42, 1152–1155 (2003). 10.1002/anie.200390303 12640647

[b14] Karagiannidis S., Mantzaras J., Bombach R., Schenker S. & Boulouchos K. Experimental and numerical investigation of the hetero-/homogeneous combustion of lean propane/air mixtures over platinum. Proc. Combust. Inst. 32, 1947–1955 (2009).

[b15] Reinke M. . High-pressure catalytic combustion of methane over platinum: *in situ* experiments and detailed numerical predictions. Combust. Flame 136, 217–240 (2004).

[b16] Schneider A. . Laser induced fluorescence of formaldehyde and Raman measurements of major species during partial catalytic oxidation of methane with large H_2_O and CO_2_ dilution at pressures up to 10 bar. Proc. Combust. Inst. 31, 1973–1981 (2007).

[b17] Zheng X., Mantzaras J. & Bombach R. Kinetic interactions between hydrogen and carbon monoxide oxidation over platinum. Combust. Flame 161, 332–346 (2014).

[b18] Karpetis A. N., Settersten T. B., Schefer R. W. & Barlow R. S. Laser imaging system for determination of three-dimensional scalar gradients in turbulent flames. Opt. Lett. 29, 355–357 (2004). 14971751 10.1364/ol.29.000355

[b19] Svanberg S. Medical diagnostics using laser-induced fluorescence. Phys. Script. T19b, 469–475 (1987).

[b20] Aldén M., Bood J., Li Z. & Richter M. Visualization and understanding of combustion processes using spatially and temporally resolved laser diagnostic techniques. Proc. Combust. Inst. 33, 69–97 (2011).

[b21] Kohse-Höinghaus K. Laser techniques for the quantitative detection of reactive intermediates in combustion systems. Prog. Energ. Combust. 20, 203–279 (1994).

[b22] Gudmundson F., Fridell E., Rosén A. & Kasemo B. Evaluation of OH desorption rates from Pt using spatially-resolved imaging of laser-induced fluorescence. J. Phys. Chem-Us. 97, 12828–12834 (1993).

[b23] Gudmundson F. . OH gas phase chemistry outside a Pt catalyst. J. Catal. 179, 420–430 (1998).

[b24] Försth M., Eisert F., Gudmundson F., Persson J. & Rosén A. Analysis of the kinetics for the H_2_+1/2O_2_ <=> H_2_O reaction on a hot Pt surface in the pressure range 0.10-10Torr. Catal. Lett. 66, 63–69 (2000).

[b25] Su H. & Yeung E. S. High-throughput screening of heterogeneous catalysts by laser-induced fluorescence imaging. J. Am. Chem. Soc. 122, 7422–7423 (2000).

[b26] Kirby B. J. & Hanson R. K. Imaging of CO and CO_2_ using infrared planar laser-induced fluorescence. Proc. Combust. Inst. 28, 253–259 (2000).

[b27] Li Z. S., Rupinski M., Zetterberg J. & Aldén M. Mid-infrared PS and LIF detection of CH_4_ and C_2_H_6_ in cold flows and flames at atmospheric pressure. Proc. Combust. Inst. 30, 1629–1636 (2005).

[b28] Kirby B. J. & Hanson R. K. Planar laser-induced fluorescence imaging of carbon monoxide using vibrational (infrared) transitions. Appl. Phys. B Lasers O 69, 505–507 (1999).

[b29] Zetterberg J. . An *in situ* set up for the detection of CO_2_ from catalytic CO oxidation by using planar laser-induced fluorescence. Rev. Sci. Instrum. 83, 053104 (2012). 22667599 10.1063/1.4711130

[b30] Blomberg S. . *In Situ* X-ray photoelectron spectroscopy of model catalysts: at the edge of the gap. Phys. Rev. Lett. 110, 117601 (2013). 25166577 10.1103/PhysRevLett.110.117601

[b31] Skoglundh M. & Fridell E. Strategies for enhancing low-temperature activity. Top. Catal. 28, 79–87 (2004).

[b32] Bourane A. & Bianchi D. Oxidation of CO on a Pt/Al_2_O_3_ catalyst: from the surface elementary steps to light-off tests I. Kinetic study of the oxidation of the linear CO species. J. Catal. 202, 34–44 (2001).

[b33] Oran U. & Uner D. Mechanisms of CO oxidation reaction and effect of chlorine ions on the CO oxidation reaction over Pt/CeO_2_ and Pt/CeO_2_/gamma-Al_2_O_3_ catalysts. Appl. Catal. B Environ. 54, 183–191 (2004).

[b34] Gustafson J. . High-energy surface X-ray diffraction for fast surface structure determination. Science 343, 758–761 (2014). 24482118 10.1126/science.1246834

[b35] Li Z. S., Rupinski M., Zetterberg J., Alwahabi Z. T. & Aldén M. Mid-infrared polarization spectroscopy of polyatomic molecules: detection of nascent CO_2_ and H_2_O in atmospheric pressure flames. Chem. Phys. Lett. 407, 243–248 (2005).

