# Supplementary material for: Spatially and temporally resolved gas distributions around heterogeneous catalysts using infrared planar laser-induced fluorescence
Source: Nat Commun. 2015 May 8;6:7076. doi: 10.1038/ncomms8076 (PMC4432634; doi:10.1038/ncomms8076)
Supplement: Supplementary Figures, Supplementary Methods and Supplementary References. — Supplementary Figures 1-3, Supplementary Methods and Supplementary References. [file ncomms8076-s1.pdf]

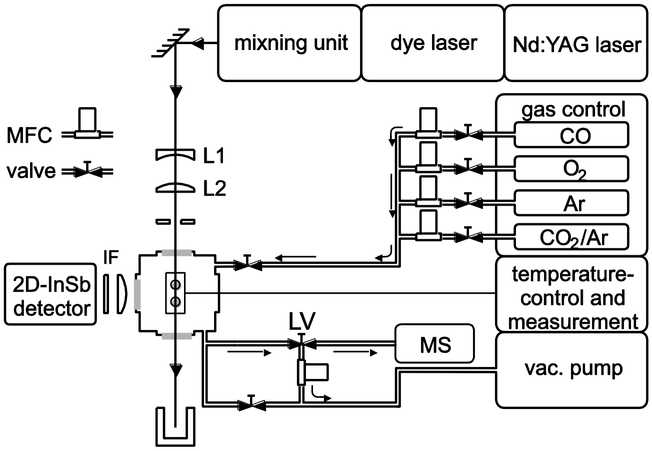

Supplementary Figure 1. Schematic of the experimental setup, the laser beam is formed into a laser sheet by two CaF_2 lenses (L1 is a negative cylindrical lens (f = -40 mm) and L2 a spherical lens (f = 250 mm)), the sheet is centered above the samples, the detector is placed at 90° with respect to the laser sheet to collect the LIF-signal which passes through an interference filter (IF). The gases are fed into the cell by mass flow controllers, and the pressure regulated by a pressure controller. All the gas that exits the reactor passes through the MS via a leak valve (LV).

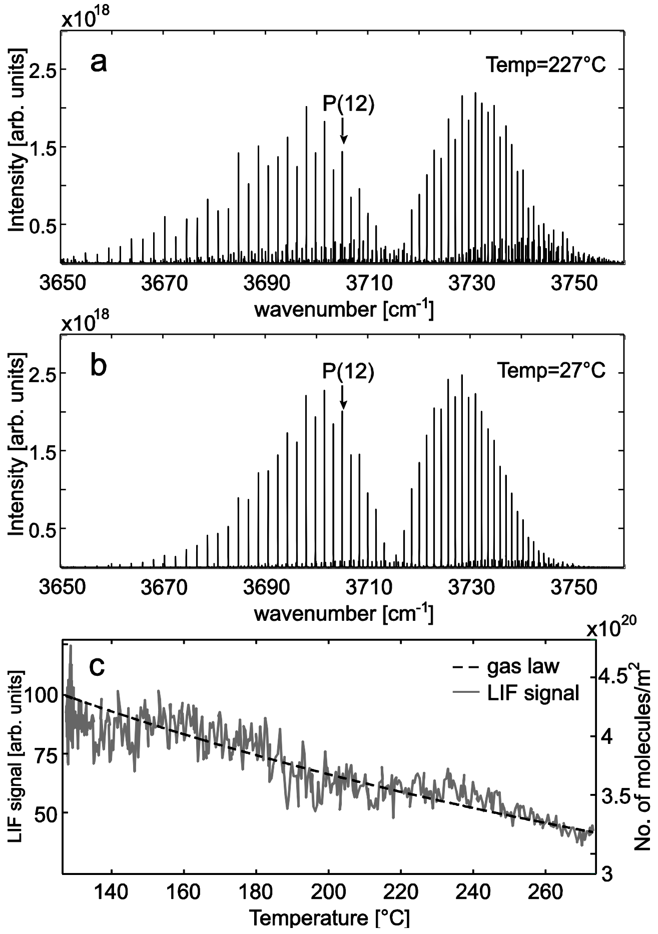

Supplementary Figure 2. Simulated absorption spectra and temperature dependence for CO_2, a) and b) show the simulated absorption-spectra of CO_2 at 227°C and 27°C respectively. c) shows the detected LIF signal at 1 mm above the sample, corrected for the change in population as a function of temperature. The dashed line shows the calculated density of molecules as a function of temperature, the P(12) line was used for laser excitation.

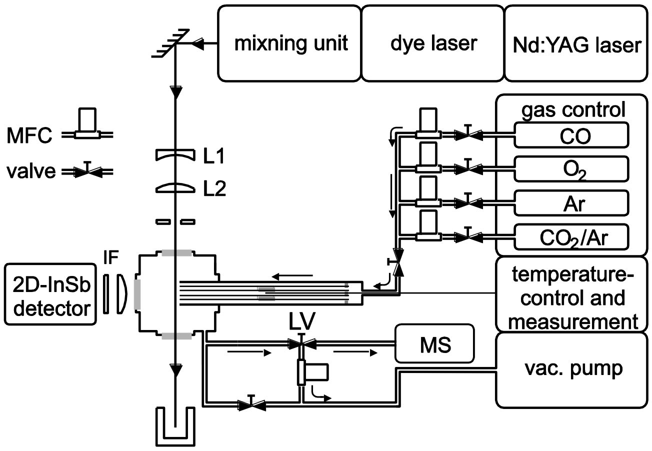

Supplementary Figure 3. A schematic of the experimental setup with the FS tubes, the laser beam is formed into a laser sheet by two CaF_2 lenses (L1 is a negative cylindrical lens (f = -40 mm) and L2 a spherical lens (f = 250 mm)), the sheet is placed as close as possible to the tube exits, the detector is placed at 90° with respect to the laser sheet to collect the LIF-signal. The gases are fed into the cell through the FS tubes and controlled by mass flow controllers, and the pressure regulated by a pressure controller. All the gas that exits the reactor passes through the MS via a leak valve (LV).

SUPPLEMENTARY METHODS

A more detailed description of laser-induced fluorescence

LIF is a common non-intrusive laser diagnostic technique for gas phase studies in many research fields[1,2], such as combustion and medical diagnostics, but is not as well-known to the catalytic community. LIF can be used to probe the temperature, velocity and concentration of a gas. The gas is probed with a laser tuned to a wavelength that perfectly matches an transition of the species of interest. If the wavelength is chosen wisely, the absorption cross section for the transition is high and no other interfering molecules will have an overlapping transition at the chosen energy. When the molecule relaxes, fluorescence light is emitted and the emitted light can then be detected. This results in an LIF signal originating from one specific species, making concentration measurements at sub-ppm levels possible.

To perform quantitative measurements with LIF is more difficult than qualitative measurements, mostly due to the collisional quenching[3]. There are several different approaches on how to quantify the LIF signal, the signal can e.g. be calibrated at known and similar conditions or the excitation can be performed in the saturated regime. It is also possible to use short-pulsed laser systems in order to quantify the collisional quenching and thereby correct for it[4].

Collisional quenching occurs when the molecules relax non-radiatively through collisions, resulting in signal loss. For the infrared (IR) spectral regime, however, where rotational (ro)-vibrational transitions are probed, the quantum yield can be described differently than for electronic transitions in the UV/visible spectral regime. This is because the vibrational-translational energy transfer, which serves to remove vibrational energy, often is slow (~1-100μs) compared to the camera integration time (~1-10μs)[5]. This is, of course, dependent on the interrogated species and its collisional partners and needs to be evaluated independently for each case and transition. In a previous study, using the same experimental set-up, the calibrated LIF signal of the CO_2 concentration over a Rh(553) single crystal was shown to be in good agreement with mass-spectrometry data[6]. In this report the aim is not to present quantitative data but to utilize the imaging capability of LIF to visualize the CO_2 distribution in the reactor with an emphasis on the extra information spatial resolution can give.

The detected signal is influenced by many physical parameters that have to be taken into account when analyzing the data. The expression for the LIF-signal S_{LIF} in the linear excitation regime is

$$S_{LIF} = \eta_c E g f(T) \sigma_0 \chi_{abs} \frac{P}{k_B T} \phi \tag{1}$$

where η_c is the experimental collection efficiency, E is the laser energy, g is a function that describes the spectral overlap between the laser and the absorption spectral lineshape, $f(T)$ is the Boltzmann fraction, σ_0 is the absorption cross section of the interrogated species, χ_{abs} is the mole fraction of the interrogated species that, together with $P/k_B T$, gives the number density of the same and ϕ is the fluorescence quantum yield. The fluorescence quantum yield, which is the fraction of excited molecules that emits fluorescence, embodies the excited-state emission rates, the collisional interaction and energy transfer between the interrogated species and the bath gas. A more detailed description of the quantum yield related to LIF in the IR spectral regime is given by Kirby et al.[5]. The expression shows that the detected LIF signal is linearly dependent on the gas density and the fraction of molecules in the state from which the laser excites the molecule, $f(T)$. The population of the energy levels in a molecule follows the Boltzmann dispersion and the signal is therefore indirect dependent on the gas temperature. Hence, determining the gas temperature requires performing and analyzing an excitation scan over the energy levels of the probed gas. Because of this temperature dependence it is desirable to choose an absorption line that is relatively insensitive (within the investigated temperature interval) when concentrations or distributions are investigated and the change should be accounted for. The present measurements were performed exciting the P12 line in the $(00^00) \rightarrow (10^001)$ transition of CO_2. In these experiments the change in the population

of the probed transition was simulated using input parameters from the HITRAN database[7], an example of a simulated ro-vibrational absorption spectra at 227°C and at 27°C of CO_2 gas is shown in Supplementary Figure 2a and b, respectively, for comparison. The signal, however, is also proportional to the number density of the probed gas. The number density, *N,* can be described by the mole fraction and the ideal gas law according to,

$$N = \chi_{abs} \frac{P}{k_B T} \quad (2)$$

where P is the total pressure, k_B the Boltzmann coefficient and T the temperature of the gas. From the expression above it can be seen that the signal will decrease as a function of temperature. In order to analyze the influence of temperature in the present measurements an experiment was carried out, where the LIF signal was collected during an increase of the temperature of the sample holder, at a constant pressure and with a known concentration of CO_2. The total pressure was regulated and kept constant by a pressure controller (Bronkhorst EL-PRESS) at 136 mbar with a continuous flow of CO_2 and Ar through the reactor. The LIF signal was recorded at 10 Hz while the temperature of the sample was increased from 120°C to 280°C. Supplementary Figure 2c shows the theoretically calculated density of molecules/m^3 (dashed line) from the ideal gas law where the gas is assumed to have the same temperature as the sample, together with the measured LIF signal (gray solid line) 1 mm above the sample surface. The LIF signal is corrected for the change in population due to the increase in temperature using data from the HITRAN database[7]. It can be seen that when both the dependence on population (for the LIF signal) and density as a function of temperature is taken into account the signal behavior can be well reproduced.

SUPPLEMENTARY REFERENCES

1 Svanberg, S. Medical Diagnostics Using Laser-Induced Fluorescence. *Phys Scripta* **T19b**, 469-475, (1987).

2 Aldén, M., Bood, J., Li, Z. & Richter, M. Visualization and understanding of combustion processes using spatially and temporally resolved laser diagnostic techniques. *Proceedings of the Combustion Institute* **33**, 69-97, (2011).

3 Kohse-Höinghaus, K. Laser Techniques for the Quantitative Detection of Reactive Intermediates in Combustion Systems. *Prog Energ Combust* **20**, 203-279, (1994).

4 Ehn, A., Johansson, O., Arvidsson, A., Aldén, M. & Bood, J. Single-laser shot fluorescence lifetime imaging on the nanosecond timescale using a Dual Image and Modeling Evaluation algorithm. *Opt Express* **20**, 3043-3056, (2012).

5 Kirby, B. J. & Hanson, R. K. Imaging of CO and CO_2 using infrared planar laser-induced fluorescence. *Proceedings of the Combustion Institute* **28**, 253-259, (2000).

6 Zetterberg, J. *et al.* An in situ set up for the detection of CO_2 from catalytic CO oxidation by using planar laser-induced fluorescence. *Rev Sci Instrum* **83**, 053104, (2012).

7 Rothman, L. S. *et al.* The HITRAN 2008 molecular spectroscopic database. *J Quant Spectrosc Ra* **110**, 533-572, (2009).
